# Editorial: Elevated Carbon Dioxide Sensing and Physiologic Effects

**DOI:** 10.3389/fphys.2022.894222

**Published:** 2022-04-27

**Authors:** Eoin P. Cummins, Ankit Bharat, Jacob I. Sznajder, István Vadász

**Affiliations:** ^1^ School of Medicine and Conway Institute of Biomolecular and Biomedical Research, University College Dublin, Dublin, Ireland; ^2^ Division of Thoracic Surgery, Feinberg School of Medicine, Northwestern University, Chicago, IL, United States; ^3^ Division of Pulmonary and Critical Care Medicine, Feinberg School of Medicine, Northwestern University, Chicago, IL, United States; ^4^ Department of Internal Medicine, Member of the German Center for Lung Research (DZL), Justus Liebig University, Universities of Giessen and Marburg Lung Center (UGMLC), Giessen, Germany; ^5^ The Cardio-Pulmonary Institute (CPI), Giessen, Germany; ^6^ Institute for Lung Health (ILH), Giessen, Germany

**Keywords:** hypercapnia, alveolar epithelium, skeletal muscle, ubiquitination, carbamate

Hypercapnia, an elevated level of carbon dioxide (CO_2_) in blood and tissue, is similarly to hypoxemia a fundamental gas exchange disturbance and is a hallmark of various pulmonary diseases (Vadász et al., 2012). Although hypoxia and hypoxemia have been very much in the focus of biological and clinical research in the last decades, much less attention has been afforded to hypercapnia. It is well known that CO_2_ is a product of cellular oxidative respiration, and is primarily eliminated in mammals by the lungs. CO_2_ homeostasis is indispensable for life, whereas alterations in CO_2_ concentrations are sensed by cells, initiating specific signaling events (Cummins et al., 2020). In this Research Topic contributed by an international consortium of researchers, in forms of original research manuscripts, reviews and perspectives, several novel or most recently discovered aspects of CO_2_ sensing and signaling mechanisms are covered. Here, we briefly summarize some of the main findings and themes discussed in the articles and outline some potential future directions for the field.

## Original Research Papers

Gabrielli et al. presented a study on the mechanisms underpinning the endocytosis of the Na,K-ATPase *via* the ubiquitin pathway. The authors using buffered CO_2_ conditions equivalent 110 mmHg demonstrated TRAF2-dependent ubiquitination of lysine 5 and 7 on the β-subunit of the Na,K-ATPase under conditions of elevated CO_2_, downstream of PKC phosphorylation. TRAF2 is well known as an E3-ubiquitin ligase capable of facilitating K63 polyubiquitination, and also as a ubiquitin ligase important for NF-kappaB-dependent signaling cascades (Zhou et al., 2013). These findings linking hypercapnia and an E3-ubiquitin ligase are of particular interest given the recent findings by the Cann group in Durham University implicating ubiquitin itself as a direct target for post-translational carboxylation (Linthwaite et al., 2021).

Ceco et al. focused their research on the molecular mechanisms underpinning skeletal muscle function. Work from the Sznajder group has linked hypercapnia with airway smooth muscle and skeletal muscle dysfunction e.g., impaired function, differentiation, regeneration and recovery (Jaitovich et al., 2015; Shigemura et al., 2018; Shigemura and Sznajder, 2021). In this study, the authors investigated the metabolic phenotype of muscle exposed to hypercapnia and identified metabolic changes resulting in elevated fatty acid oxidation. Interestingly, use of an inhibitor of fatty acid shuttling, etomoxir, reversed the CO_2_-dependent phenotype. The authors proposed that hypercapnia “overloads” mitochondrial metabolism in skeletal muscle. Further work should reveal whether this metabolic phenotype unique to skeletal muscle or is more universal.

Üçpunar et al. focused their work on CO_2_ sensitivity and signaling in Drosophila. While Drosophila-based studies on CO_2_ sensitivity and sensing have traditionally focused on attraction to rotting fruit or elevated CO_2_ (van Breugel et al., 2018), instead this study focused on the response of Drosophila to current ambient atmospheric CO_2_ (400 ppm) compared to CO_2_-free air. Evidence from this study suggests that ambient levels of CO_2_ are sensed, and that the flies have a preference for levels of CO_2_ that are lower than our current environment. Thus, this paper raised the intriguing question as to how an odor (CO_2_) can be both attractive and aversive in the same animal. As part of the explanation, the authors proposed a circuit dependent on GR63a/GR21a for environmental preference to sub-atmospheric CO_2_ levels.

## Literature Reviews and Perspectives

Kryvenko et al. reviewed the role of the endoplasmic reticulum (ER) as a central signaling organelle in the context of CO_2_-dependent signal transduction (Jaitovich et al., 2015). Recent evidence suggest that elevated Ca^2+^ downstream of hypercapnia may derive from the ER. Normal ER function is reliant on sufficient ATP and consequently hypercapnia-dependent metabolic changes can impinge on normal ER function and potentially lead to ER stress (Depaoli et al., 2019; Kryvenko et al., 2020). Dysregulation of Ca^2+^ and ATP-dependent chaperones can thus potentially contribute to aberrant protein folding in the ER under conditions of hypercapnia. Furthermore, the authors delineated the interplay between potentially maladaptive cellular responses to CO_2_ in the context of the ER.

Shigemura et al. reviewed the impact of CO_2_-dependent gene expression in different tissues, with a focus on the lung, skeletal muscle and innate immune signaling. The authors highlighted that many of the observed responses to hypercapnia are maladaptive, discussing recent clinical trial data in patients with severe lung diseases (Nin et al., 2017). It is clear that certain tissue types elicit signaling in a tissue-specific manner e.g., MuRF-1 is a key player in skeletal muscle dysfunction but is specific to that tissue type (Files et al., 2012). Interestingly, MuRF-1 is an E3 ligase like TRAF-2 and suggests that the ubiquitin proteasome system may be a common signaling thread in the response to CO_2_. Another common and conserved signaling component involved in the cellular response to CO_2_ is the Wnt signaling pathway, which is highly conserved in animals and has been shown to be sensitive to CO_2_ across species (Shigemura et al., 2019).

Blake and Cann reviewed the role of carbamylation, a post-translational modification at N-terminal α-amino groups or lysine ɛ-amino groups of target proteins by CO_2_. This direct interaction of proteins and CO_2_ may alter protein function and might play a role in CO_2_ sensing and initiation of subsequent signaling events. In particular, the review discusses the regulation of several proteins by carbamate post-translational modification as well as novel methodological advances in uncovering protein carbamylation at the level of the proteome. Among others, the manuscript details the role of carbamylation in the mechanism by which CO_2_ alters affinity of hemoglobin for oxygen [Bohr effect; reviewed in (West, 2019)], thereby linking carbamate post-translational modification to impaired gas exchange. Other examples of proteins functions of which are altered by carbamylation discussed in the review include, ribulose 1,5-bisphosphate carboxylase-oxygenase, which is responsible for atmospheric CO_2_ elimination during photosynthesis (Andersson and Backlund, 2008), bacterial β-lactamases that enable resistance to β-lactam antibiotics as well as ureases, which catalyze hydrolysis of urea to carbonic acid and ammonia with relevance to human disease. Moreover, the regulation of connexin 26, a transmembrane protein that form hexameric connexons of gap junctions or hemichannels and which undergo conformational changes upon carbamylation is described, thus identifying a potential CO_2_ sensing molecule.

Balnis et al. built on existing limitations of certain animal models to develop a new paradigm where animals demonstrate chronic obstructive pulmonary disease (COPD)-driven muscle dysfunction, but exhibit normal pCO_2_ levels. Thus, hypercapnia can be superimposed on this model to examine the cross talk between the distinct stimuli. Interestingly, the authors reported a relatively “stepwise” decrease in absolute force generation with COPD, hypercapnia and COPD + hypercapnia, in contrast to specific force generation where a synergistic reduction in force was observed when COPD and hypercapnia were combined. The authors highlighted that these data underlie a complex phenotype that may relate to hypercapnia-dependent differences in ECM gene expression in the murine COPD model.

## Conclusion and Future Directions

Taken together, this Research Topic on “*Elevated Carbon Dioxide Sensing and Physiologic Effects*” addresses some of the state of the art topics in the field. How is CO_2_ sensed acutely and more chronically? What are the mechanisms underpinning these responses e.g., neuronal receptors, signaling cascades, post-translational mechanisms and transcription factors? What are the consequences of these signaling mechanisms for the organism? Is the response to elevated CO_2_ adaptive or maladaptive or are these consequences context-specific? Is the organismal response to CO_2_ dependent on other factors such as the severity and nature of disease? This is relevant to the current coronavirus disease (COVID-19) pandemic as hypercapnia is often an inevitable clinical consequence of lung-protective mechanical ventilation strategies to reduce the incidence of ventilator-induced lung injury in patients with acute respiratory distress syndrome (ARDS). The relatively long duration of mechanical ventilation in patients with COVID-19 compared with patients with other causes of ARDS [median ∼14 compared with ∼4 days (Grant et al., 2021)] increases the duration of exposure to hypercapnia and thus has deleterious effects on lung repair after injury. Based on this Research Topic, a strong argument is made for 1) further examination of the ER as a central signaling organelle in hypercapnia and 2) altered ubiquitin-dependent signaling. The cross-talk between these two elements is worthy of future research, particularly in conjunction CO_2_-dependent cellular metabolic changes, which can influence both pathways and are of clinical relevance to patients with ARDS and COVID-19. The CO_2_-field is ripe for attracting further excellent research in this area.
